# The Effects of a Combined Exercise Intervention on Body Composition, GDF-15, Apelin-12, and IL-15 Among Older Korean Women According to Obesity Status

**DOI:** 10.3390/jcm14144981

**Published:** 2025-07-14

**Authors:** Jeongsook Kim, Eadric Bressel, Minkyo Kim, Taekyu Kim, Suhan Koh, Doyeon Kim

**Affiliations:** 1 Exercise Physiology Laboratory, Department of Physical Education, Pusan National University, Busan 46241, Republic of Korea; 2 Department of Kinesiology and Health Science, Utah State University, Logan, UT 84322, USA

**Keywords:** combined exercise, obese, older women, body composition, GDF-15, apelin-12, IL-15

## Abstract

**Background:** The purpose of this study was to investigate the effects of a 16-week exercise program combining aerobic and resistance training on body composition, growth differentiation factor-15 (GDF-15), apelin-12, and interleukin-15 (IL-15) in older Korean women according to obesity status. **Methods**: Participants were divided into obesity (*n* = 15) and normal-weight groups (*n* = 14). A walking exercise was performed at 60–70% heart rate reserve (RPE 13–15). The bodyweight resistance exercises were progressively intensified over 16 weeks. Analysis methods included two-way repeated measures ANOVA, ANCOVA, and paired and independent *t*-tests. **Results**: Significant main effects of time and group were observed in body weight (*p* < 0.001), and both groups demonstrated significant within-group reductions in body mass index (BMI) (obese: *p* < 0.001; normal-weight: *p* < 0.05), along with significant between-group differences (*p* < 0.001). The percentage of body fat significantly decreased over time (*p* < 0.01) and differed between groups (*p* < 0.001). GDF-15 exhibited a significant group × time interaction (*p* < 0.05) and a main group effect (*p* < 0.05). Although no statistically significant changes were observed in Apelin-12 levels, an opposite trend was identified between groups, with an increase in the obese group and a decrease in the normal-weight group. For IL-15, no significant interaction effect was found between the groups. **Conclusions**: The 16-week combined exercise intervention improved key markers of body composition, particularly in obese older women, and led to increased GDF-15, indicating potential metabolic benefits. While changes in apelin-12 and IL-15 were not statistically significant, the findings support the utility of combined exercise for mitigating fat accumulation and promoting healthy aging in older adults.

## 1. Introduction

Obesity, beyond its metabolic implications, has been increasingly linked to an elevated risk of cancer due to chronic, low-grade inflammation initiated by dysfunctional adipose tissue. This pro-inflammatory environment arises from shifts in the endocrine functions of fat tissue, particularly the secretion of biologically active mediators such as adipokines, cytokines, and hormones [1]. Among these, adipokines including apelin and interleukins (ILs) are known to be pivotal in driving immune responses that influence both local and systemic inflammation [2].

Emerging evidence suggests that exercise acts as a powerful modulator of these molecular signals. Apelin, a peptide secreted by both adipose and muscle tissue, has garnered attention for its role in muscle adaptation. It is upregulated following physical activity and is associated with improved mitochondrial function, muscle fiber regeneration, and hypertrophic response. Additionally, its regulatory role extends to modulating thermogenic pathways by enhancing brown adipose tissue activity and mitigating the deleterious effects of sarcopenic obesity [3]. Resistance training, in particular, has been shown to increase both IL-15 and apelin levels, contributing to anti-inflammatory adaptations [3].

Circulating apelin concentrations respond favorably to both acute and chronic exercise stimuli, with obese individuals showing greater sensitivity to exercise-induced increases compared to non-obese counterparts [4]. In older adults, structured physical activity interventions, such as stair climbing, have been found to significantly elevate plasma apelin levels, highlighting its relevance in age-related metabolic resilience and muscle preservation [5].

Another molecule of interest is growth differentiation factor-15 (GDF-15), which plays a central role in regulating appetite and energy balance. As a stress-responsive cytokine, GDF-15 is upregulated during aging and metabolic dysfunction, and its elevation through exercise has been linked to appetite suppression, improved glycemic control, and reductions in fat mass [6,7,8]. Given its multifaceted functions, GDF-15 is emerging as a viable target for non-pharmacological obesity interventions.

IL-15 is abundantly expressed in skeletal muscle and plays a key role in promoting muscle hypertrophy by increasing in response to exercise [9]. Genetic polymorphisms in IL-15 have been correlated with variability in response to resistance training, suggesting individual differences in exercise adaptability [10]. Though baseline IL-15 levels are often diminished in aged or obese individuals, exercise—both aerobic and resistance—temporarily elevates its expression, potentially reversing adverse body composition trends [11]. In aged or obese mammals, circulating IL-15 levels are typically low, but they increase transiently after exercise. Furthermore, IL-15 has the potential to inhibit adipocyte differentiation and reverse diet-induced obesity [12].

Combined exercise protocols that integrate aerobic and resistance modalities have shown consistent benefits in improving anthropometric parameters such as body mass index (BMI), body fat percentage (%BF), and waist circumference, while also enhancing cardiovascular fitness [13]. Specifically, in older women with obesity, a 12-week regimen has demonstrated significant improvements in lean body mass and physical activity levels, suggesting that tailored interventions can combat both sarcopenia and obesity [14].

Taken together, these findings support the integration of multidimensional exercise strategies to target key molecular pathways involved in aging and obesity. Therefore, the present study aims to examine the effects of a 16-week moderate-to-high-intensity combined exercise program on body composition and circulating levels of GDF-15, apelin-12, and IL-15 in older women with obesity, contributing to the growing understanding of exercise-induced molecular adaptations in this population.

## 2. Materials and Methods

### 2.1. Study Participants

**Figure 1:**
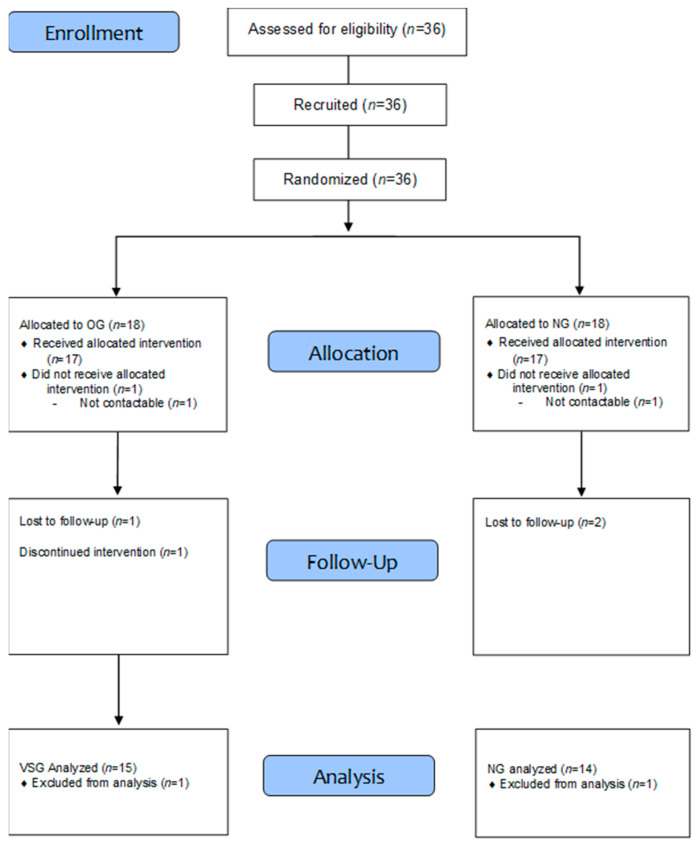
Procedures of this study.

The sample size required for this study was calculated using the G*Power 3.1 program (Heinrich-Heine University Düsseldorf, Düsseldorf, Germany) based on a repeated measures ANOVA. With an effect size of f = 0.28, two groups, two measurements, a power of 0.8, and a significance level of 0.05, the required sample size was 28 participants. Accounting for an estimated dropout rate of approximately 25%, the final sample size was increased to 36 participants. Participants were older women, aged 70–85 years, residing in G city, who did not engage in regular exercise; they were categorized into obese (*n* = 18) and normal-weight (*n* = 18) groups, with obesity defined as BMI ≥ 25 kg/m^2^ and %BF ≥ 30% [15]. They were divided by simple random sampling. After excluding those who dropped out for personal reasons or had unreliable measurement and test results, the final analysis included 29 participants: 15 from the obesity group and 14 from the normal-weight group. Before the experiment, approval was obtained from the Institutional Review Board of Pusan National University (PNU IRB/2023_85_HR). The study’s purpose and objectives were thoroughly explained to participants, and informed consent was obtained from those who voluntarily agreed to participate. The study procedures and participants’ physical characteristics are presented in Table 1 and Figure 1.

### 2.2. Exercise Program

**Figure 2:**
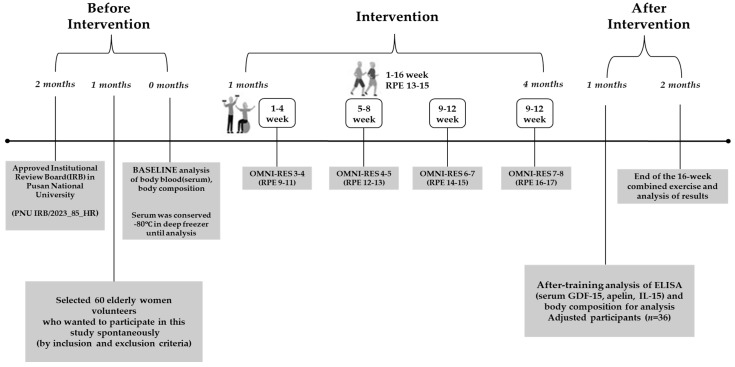
Flowchart of exercise intervention.

The combined exercise program we tested was a modified and refined version of the program developed by Hyun et al. [16]. It consisted of 60 min sessions, conducted three times per week for 16 weeks. To ensure participant safety, exercise intensity was gradually increased throughout the intervention period in accordance with guidelines published by the American College of Sports Medicine [17]. Walking intensity was maintained at 60–70% of heart rate reserve (RPE 13–15) and monitored using a wireless heart rate monitor (Polar System, Kempele, Finland). Bodyweight resistance exercises were performed using the OMNI-Resistance Exercise Scale (OMNI-RES), a 10-point rating system for resistance exercise intensity. During weeks 1 to 4, exercises were performed at low intensity (OMNI-RES 3–4); during weeks 5 to 8, at low-to-moderate intensity (OMNI-RES 4–5); during weeks 9 to 12, at moderate intensity (OMNI-RES 6–7); and during weeks 13 to 16, at high intensity (OMNI-RES 7–8) [18]. The detailed procedures of the combined exercise program are presented in Figure 2.

### 2.3. Measurement Items and Analysis Methods

#### 2.3.1. Body Composition

Before the assessment, participants removed any metal objects. Height was measured using an automatic stadiometer (DS-103M, Jenix Co., Daegu, Republic of Korea). Body weight (kg), body fat mass (kg), skeletal muscle mass (kg), and %BF were measured using a body composition analyzer (InBody 770, Biospace Co., Seoul, Republic of Korea). Before undergoing InBody measurements, the subjects refrained from engaging in moderate- to high-intensity exercise for 12 h and avoided consuming food or beverages for 4 h.

#### 2.3.2. Blood Analysis

Blood samples were collected between 8:00 and 10:00 a.m. after participants had fasted for at least 8 h, starting from 9:00 p.m. the previous night. A clinical laboratory technician performed the venipuncture, drawing 10 mL of blood from the forearm vein using a vacuum blood collection tube. The collected blood was placed in a serum separator tube and centrifuged at 3000 rpm for 20 min using a Combi-514 centrifuge (Hanil, KOR). After serum separation, the supernatant was transferred to a 1.5 mL microtube and stored at −80 °C until analysis.

GDF-15 levels were measured using the Human GDF-15 Quantikine ELISA Kit (R&D Systems, Minneapolis, MN, USA) and analyzed at an absorbance of 450 nm with a Microplate Reader AMR-100 (Allsheng, Hangzhou, China). Apelin-12 levels were measured using the Apelin (Human, Rat, Mouse, Bovine) Extraction-Free EIA Kit (Phoenix Pharmaceuticals, Burlingame, CA, USA) and analyzed at 450 nm absorbance using a Microplate Reader AMR-100 (Allsheng, Hangzhou, China). IL-15 levels were measured using the Human IL-15 Quantikine ELISA Kit (R&D Systems, USA) and analyzed at an absorbance of 450 nm with a Microplate Reader AMR-100 (Allsheng, Hangzhou, China).

### 2.4. Data Processing

Data were processed using SPSS version 27.0. The mean (M) and standard deviation (SD) for each measurement were calculated. Additionally, the Shapiro–Wilk test (for small sample sizes) was performed to verify the normality of the data before analysis. The interaction between groups and time for each variable was assessed using a two-way repeated measures ANOVA. To account for the homogeneity of pretest values, analysis of covariance (ANCOVA) was performed to evaluate the main intergroup effects. Mean intra- and intergroup differences before and after exercise were assessed using paired and independent *t*-tests, respectively. The significance level for all tests was set at 0.05. This study did not apply any correction for multiple comparisons.

## 3. Results

### 3.1. Body Composition

Table 2 presents the effects of the 16-week combined exercise intervention on body composition variables, including body weight, body mass index (BMI), and percentage of body fat (%BF), according to obesity status.

For body weight, no significant group × time interaction was found; however, significant main effects were observed for both time and group (*p* < 0.001). Within-group comparisons revealed significant reductions in body weight for both the obesity group (*p* < 0.001) and the normal-weight group (*p* < 0.05). Between-group differences were significant at both pretest and posttest (*p* < 0.001), though no significant difference in the rate of change was noted.

Similarly, for BMI, there was no significant interaction effect, but significant main effects of time and group were found (*p* < 0.001). BMI decreased significantly over time in both the obesity (*p* < 0.001) and normal-weight (*p* < 0.05) groups, with significant between-group differences at both pre- and post-intervention (*p* < 0.001). Again, the rate of change did not differ significantly between groups.

For %BF, no group × time interaction was detected; however, significant main effects of time (*p* < 0.01) and group (*p* < 0.001) were identified. The obesity group showed a significant reduction over time (*p* < 0.05), and both pretest and posttest values differed significantly between groups (*p* < 0.001). As with other variables, the rate of change was not significantly different between the groups.

### 3.2. GDF-15

The changes and interactions within and between groups for GDF-15 are presented in Table 3. An interaction effect between group and time was observed (*p* < 0.05), and a main effect between the groups was found (*p* < 0.05). However, no main effect was observed for time. No significant differences were found within the groups for changes between timepoints or between groups for pre- and posttest, although a significant difference in change rates was observed between the obesity and normal-weight groups from pre- to posttest (*p* < 0.05).

### 3.3. Apelin-12

Table 4 presents the results for apelin-12 levels. No significant group × time interaction was observed, and no main effects of time or group were detected. Within-group comparisons revealed no significant changes over time in either the obesity or normal-weight groups. Similarly, no significant differences were observed between groups at either the pretest or posttest. Furthermore, the rate of change from pre- to post-intervention did not differ significantly between the groups.

### 3.4. IL-15

The changes within groups, between groups, and the interaction effects for IL-15 are presented in Table 5. No time × group interaction effects were observed. However, a significant main effect of time was found (*p* < 0.05), though no significant differences were found in changes within groups over time, nor in the pre- and posttest differences between groups. Additionally, there was no significant difference in the change rates between the obesity and normal-weight groups.

## 4. Discussion

### 4.1. Body Composition

The significant reduction in BMI and percentage body fat (%BF) observed in the obese group, along with the maintenance of %BF in the normal-weight group, aligns closely with the American College of Sports Medicine (ACSM) guidelines for exercise programming aimed at weight loss in individuals with obesity [17]. These guidelines prioritize sustained moderate-intensity aerobic exercise and recommend the inclusion of resistance training. The results of the present study reflect the efficacy of such a combined exercise approach.

In particular, for individuals with a BMI ≥ 30 kg/m^2^, interventions involving both aerobic and resistance exercises have been shown to effectively reduce body weight, waist circumference (WC), and %BF, while simultaneously improving cardiorespiratory fitness (CRF). These findings support the notion that both aerobic and resistance training modalities contribute significantly to improvements in aerobic capacity, blood lipid profiles, and insulin sensitivity. Additionally, in obese adults, this combined exercise strategy may enhance leptin production, which is associated with decreased adipose tissue accumulation [13].

Park et al. [19] reported that a 12-week regular combined exercise regimen in sedentary obese older adults significantly improved body composition, cardiovascular risk factors, blood pressure, arterial stiffness, and overall physical function. These results underscore the positive impact of concurrent aerobic and resistance exercise on reducing body weight and body fat in obese older women. Moreover, the observed improvements in body composition, blood pressure, and arterial stiffness, along with enhanced physical functioning, highlight the potential of combined exercise interventions in preventing and managing obesity-related metabolic disorders in the elderly.

Given that older adults, especially those aged 65 and over, demonstrate low levels of physical activity—with participation rates further declining with age [20], promoting regular physical activity is critical. Physical activity not only mitigates the physiological decline in exercise capacity associated with aging but also supports optimal body composition, psychological well-being, and the management of chronic diseases. It has also been linked to reduced risk of physical disability and increased longevity [17]. For older adults at risk of chronic diseases due to obesity, reducing sedentary behavior and promoting an active lifestyle leads to a range of health benefits, including improved physical health, enhanced energy, greater self-confidence, and reduced stress [20]. In this context, the improvements in body composition observed through combined exercise in the present study are particularly meaningful.

Therefore, this study confirms the positive impact of combined exercise on body composition as a preventive and therapeutic measure for obesity, particularly in older adults. Both the obese and normal-weight groups demonstrated changes in body composition—most notably, a decrease in BMI. While the obese group exhibited significant reductions in %BF, the normal-weight group maintained their BMI and %BF within the normal range post-intervention. These findings indicate that combined aerobic and resistance training is highly effective in promoting weight loss and reducing fat mass in obese older women. Moreover, the role of exercise in maintaining healthy body composition is evident for both obese and normal-weight individuals.

### 4.2. GDF-15

Adipose tissue plays a critical role in regulating energy homeostasis, insulin sensitivity, and both lipid and carbohydrate metabolism, thereby contributing to the development of insulin resistance in obesity [21]. In a study investigating the effect of weight loss interventions on serum levels of growth differentiation factor-15 (GDF-15) and its relationship with metabolic outcomes, Cai et al. [22] found that three weeks of moderate-to-high-intensity exercise in overweight and obese adults resulted in reductions in body weight, fat mass, and waist circumference. Notably, 77.3% of participants exhibited increased levels of GDF-15.

GDF-15, a stress-responsive cytokine, has thus emerged as a potential biomarker for metabolic improvement in overweight and obese individuals. In the present study, combined exercise intervention in sedentary older women resulted in increased GDF-15 levels in the obese group, whereas the normal-weight group showed a tendency for decreased GDF-15 levels. This divergence may be attributed to the initially higher baseline GDF-15 concentrations in the normal-weight group compared to the obese group, raising questions as to whether the exercise intervention itself directly influenced GDF-15 expression.

These findings partially contrast with those of Zhang et al. [8], who reported that GDF-15 levels increased acutely post-exercise in normal-weight individuals, and Wang et al. [23], who demonstrated that moderate-intensity endurance training elevated GDF-15 levels across a wide range of populations, including underweight subjects. Therefore, future research should investigate how different durations and types of exercise programs impact GDF-15 in normal-weight or underweight individuals. To draw clearer conclusions about the effect of exercise on GDF-15, future interventions will require more rigorous experimental controls.

Long-term endurance exercise has been shown to upregulate GDF-15 expression and activate its receptor complex, GFRAL-RET, which plays a role in appetite suppression, inflammation reduction, and enhanced insulin sensitivity under healthy physiological conditions [24]. As a cytokine with systemic metabolic influence, GDF-15 expression is induced by intense exercise and other stressors [6]. Recent studies indicate that individuals who exhibited the largest increases in GDF-15 also experienced improvements in whole-body fat oxidation, metabolic flexibility, and insulin sensitivity [25]. A 12-week aerobic exercise regimen performed five days per week was found to increase GDF-15 levels in individuals with obesity and was associated with fat mass reduction [8].

Moreover, aerobic exercise at 67% of VO_2_max for one hour significantly elevated circulating GDF-15 levels in healthy subjects [26], with even more pronounced increases observed following ultra-marathon events [27]. Moderate-intensity endurance and high-intensity training consistently elevated GDF-15 across diverse populations, including those who were underweight, obese, elderly, sedentary, or physically trained [23].

Another study exploring the relationship between exercise, GDF-15 levels, and weight loss in obese individuals reported that exercise-induced increases in GDF-15 were positively associated with reductions in fat mass among older adults [8]. Given the rising global prevalence of obesity and its economic and health burdens—particularly in older adults with limited treatment options—the observed increase in GDF-15 following combined exercise in this study suggests a meaningful correlation with weight and fat mass reduction.

Furthermore, GDF-15 contributes to energy balance by suppressing food intake. Therefore, more studies are warranted to clarify whether exercise-induced changes in GDF-15 mediate appetite regulation, and how exercise intensity and duration modulate these effects. Such research would emphasize the vital role of sustained physical activity in promoting healthy aging in older women and inform strategies to optimize exercise interventions for metabolic health.

### 4.3. Apelin-12

Apelin has emerged as a key regulatory peptide involved in energy metabolism, vascular homeostasis, and muscle physiology. Both apelin deficiency and excess have been associated with the acceleration of muscle aging and tissue damage mediated through inflammatory responses, thus contributing to the progression of disease states. Particularly in obese elderly women, apelin may function as a sensitive biomarker of metabolic health [2,23,28]. Skeletal muscle contraction induced by exercise has been shown to stimulate apelin secretion into circulation, and elevated apelin expression has been proposed as a physiological indicator of improved health outcomes in older adults [28].

The current study hypothesized that a 16-week combined exercise program would significantly increase serum apelin-12 levels in elderly women. However, the findings did not reveal statistically significant changes in either the obese or normal-weight groups. Notably, baseline apelin levels were higher in the obese group compared to the normal-weight group. Following the intervention, apelin levels declined slightly in the normal-weight group, whereas no consistent directional change was observed in the obese group. Across both groups, there was considerable individual variability in apelin response post-intervention, suggesting that responsiveness may depend on intrinsic factors such as baseline metabolic status, exercise sensitivity, or health condition.

Previous research by Sheibani et al. [29] reported significant increases in both plasma apelin and insulin concentrations following aerobic training in obese women, highlighting the physiological benefits of exercise on vascular function and metabolism, which contribute to cardiovascular protection and fat mass reduction. Similarly, Ji et al. [30] emphasized that apelin responses to exercise in older women varied depending on baseline metabolic health, exercise intensity, and body composition, including BMI. Fujie et al. [31] further demonstrated that high-intensity or prolonged aerobic training elicited greater elevations in apelin-12 levels, which were strongly associated with reductions in fat mass and enhancements in cardiovascular function.

Given that apelin secretion is stimulated via muscle contraction and circulatory flow, the absence of significant changes in the present study may be attributed to participants’ sedentary baseline status and the relatively gradual progression in exercise intensity over the 16-week intervention. This suggests that the exercise dose may not have been sufficient to elicit measurable hormonal adaptations, especially in previously inactive obese elderly women.

Furthermore, individual variability in apelin response may be influenced by multiple factors, including age, baseline fitness, body mass, and intervention duration. Future research should incorporate longitudinal study designs, include diverse age groups and body types, and integrate molecular analyses to elucidate the mechanistic underpinnings of apelin regulation. Additionally, a systematic investigation of how apelin-12 responds to different exercise types, intensities, and frequencies could offer valuable insights for the development of targeted interventions aimed at promoting healthy aging and managing obesity.

### 4.4. IL-15

In obese elderly women, interleukin-15 (IL-15) has been identified as a critical myokine mediating the beneficial effects of exercise on skeletal muscle metabolism, adipose tissue regulation, and systemic inflammation. It is generally accepted that combined exercise interventions—integrating aerobic and resistance components—elevate IL-15 expression, thereby supporting muscle hypertrophy, fat oxidation, and anti-inflammatory responses. However, contrary to these well-established outcomes, the current study observed a reduction in IL-15 levels following a combined exercise protocol, highlighting a paradoxical response that warrants further exploration.

This unexpected decline may be attributed to several interacting factors, including the type and intensity of exercise, the presence of sarcopenia, and most notably, the nutritional status of participants. It has been suggested that under metabolic stress or energy-deficient conditions, the physiological benefits of IL-15 may be blunted, or in some cases, reversed.

The previous literature largely supports IL-15 upregulation in response to chronic exercise. For example, increased IL-15 levels were observed after 12 weeks of structured training in elderly women [32]. Similarly, short-term endurance exercise was found to be inversely correlated with visceral fat mass via IL-15 elevation in obese participants [33]. Additionally, an 8-week resistance training program significantly increased serum IL-15 concentrations in overweight women [34].

Nonetheless, reports of IL-15 reduction in response to exercise are rare, particularly among elderly or obese female populations. Consistent with the present findings, decreased IL-15 gene expression was observed in certain groups exposed to high-intensity aerobic-dominant combined exercise [35]. This reduction was attributed to excessive oxidative stress and potential energy insufficiency in skeletal muscle, underscoring that exercise does not universally promote IL-15 upregulation across all physiological conditions.

Furthermore, Kim [35] noted that inadequate protein intake may amplify exercise-induced stress, leading to IL-15 suppression rather than enhancement. These findings suggest that nutritional insufficiency in obese elderly women may create a physiological environment similar to that of energy-deficient or overtrained states, thereby altering expected myokine responses.

Several additional scenarios may contribute to IL-15 downregulation: mismatched exercise intensity and dietary intake, short or irregular training durations, underlying pathologies such as sarcopenia or metabolic syndrome, or chronic maladaptation to exercise. Taken together, these factors emphasize the necessity for individualized and periodized exercise prescriptions that account not only for training variables but also for nutritional adequacy and comorbid health conditions in this vulnerable population.

### 4.5. Limitations

Despite the meaningful findings, this study has several limitations that should be acknowledged.

First, the sample size was relatively small, and participants were recruited from a single community setting, which may limit the generalizability of the results to broader populations of older adults, particularly those with more diverse health backgrounds, socioeconomic statuses, or cultural habits.

Second, dietary intake and nutritional status were not strictly monitored or controlled throughout the 16-week intervention. Given that IL-15 and other exercise-induced myokines are sensitive to energy balance and protein availability, the lack of nutritional control may have influenced hormonal responses, especially in the observed IL-15 downregulation.

Third, although the exercise program was designed based on established guidelines, individual variability in training intensity, compliance, and recovery capacity was not fully captured. This may have led to inconsistent physiological adaptations, particularly in hormonal outcomes such as apelin-12 and IL-15.

Fourth, molecular mechanisms were inferred based on serum concentrations, but no muscle biopsies or intracellular signaling analyses were conducted to directly assess local expression of IL-15, GDF-15, or apelin within skeletal muscle tissue.

Fifth, no correction for multiple comparisons was applied. As multiple statistical tests were conducted, the risk of Type I errors may have increased. Unfortunately, appropriate correction methods for multiple comparisons were not implemented in this study.

Finally, hormonal responses were only assessed at pre- and post-intervention timepoints, missing potential transient or acute changes in cytokines like IL-15 or GDF-15, which may fluctuate rapidly following individual sessions. More frequent measurements, including immediate post-exercise sampling, would provide a clearer temporal profile of myokine dynamics.

## 5. Conclusions

This study demonstrates that a 16-week combined aerobic and resistance training program can significantly improve body composition and modulate the expression of key myokines—GDF-15, apelin-12, and IL-15—in older women, with differential responses observed based on obesity status. The findings confirm the positive impact of exercise on reducing BMI and body fat, particularly in obese older adults, aligning with international guidelines for weight management in aging populations.

Notably, the increase in GDF-15 levels in the obese group suggests that this cytokine may serve as a promising biomarker of metabolic improvement and fat mass reduction in response to exercise. However, the absence of significant changes in apelin-12 and the unexpected reduction in IL-15 highlight the complexity of individual hormonal responses and the potential influence of factors such as exercise intensity, nutritional status, and baseline physiological conditions.

These results underscore the need for personalized and integrative exercise prescriptions that consider both training parameters and nutritional support. Collectively, this study contributes to the growing body of evidence supporting the role of physical activity as a therapeutic strategy to promote metabolic health and healthy aging in older women, particularly those with obesity.

## Figures and Tables

**Table 1:** Physical characteristics of subjects in each group.

Variables Group	Age (yrs)	Height (cm)	Weight (kg)	BMI (kg/m^2^)	%BF (%)
OG (*n* = 15)	76.67 ± 4.86	151.07 ± 4.06	62.57 ± 6.41	27.37 ± 2.26	38.69 ± 4.97
NG (*n* = 14)	77.57 ± 6.12	149.61 ± 5.54	47.89 ± 7.10	21.36 ± 2.49	25.46 ± 4.35

Values are M ± SD; BI confirmMI: body mass index, %BF: percentage of body fat; OG: obesity group, NG: normal-weight group.

**Table 2:** Changes in body composition after 16-week combined exercise intervention.

Variable	Graph.	Pre	Post	Diff (%)	Paired-t	F	
Weight (kg)	OG (*n* = 15)	62.57 ± 6.41	61.43 ± 6.40	−1.83	5.056 ***	Group	33.046 ***
	NG (*n* = 14)	47.89 ± 7.10	47.06 ± 7.40	−1.79	2.244 *	Time	21.254 ***
	t-value	−5.850 ***	−5.609 ***	0.039		G × T	0.504
BMI (kg/m^2^)	OG (*n* = 15)	27.37 ± 2.26	26.87 ± 2.29	−1.83	5.149 ***	Group	44.947 ***
	NG (*n* = 14)	21.36 ± 2.50	20.97 ± 2.58	−1.86	2.368 *	Time	22.301 ***
	t-value	−6.806 ***	−6.532 ***	−0.030		G × T	0.321
%BF (%)	OG (*n* = 15)	38.69 ± 4.97	36.85 ± 6.14	−4.89	2.211 *	Group	49.166 ***
	NG (*n* = 14)	25.46 ± 4.35	24.69 ± 4.37	−2.95	1.994	Time	7.701 **
	t-value	−7.605 ***	−6.102 ***	0.704		G × T	1.314

Values are M ± SD; BMI: body mass index, %BF: percentage of body fat; OG: obesity group, NG: normal-weight group; “t” refers to the t-statistic obtained from either a paired samples *t*-test or an independent samples *t*-test; * *p* < 0.05, ** *p* < 0.01, *** *p* < 0.001.

**Table 3:** Changes in GDF-15 after 16-week combined exercise intervention.

Variable	Group	Pre	Post	*diff* (%)	Paired-t	*F*	
GDF-15 (pg/mL)	OG (*n* = 15)	1271.30 ± 375.93	1403.76 ± 435.79	132.46	−1.485	Group	4.564 *
	NG (*n* = 14)	1457.16 ± 794.08	1259.46 ± 405.17	−197.7	1.503	Time	0.173
	*t-value*	0.815	−0.922	−2.103 *		G × T	4.423 *

Values are *M* ± *SD*; GDF-15: growth differentiation factor-15; OG: obesity group, NG: normal-weight group; “t” refers to the t-statistic obtained from either a paired samples *t*-test or an independent samples *t*-test; * *p* < 0.05.

**Table 4:** Changes in apelin-12 after 16-week combined exercise intervention.

Variable	Group	Pre	Post	*diff* (%)	Paired-t	*F*	
Apelin-12 (pg/mL)	OG (*n* = 15)	251.09 ± 42.23	251.71 ± 45.30	0.62	−0.078	Group	0.548
	NG (*n* = 14)	241.18 ± 34.87	234.29 ± 48.83	−6.69	1.220	Time	0.401
	*t-value*	−0.686	−0.996	−0.758		G × T	0.575

Values are *M* ± *SD*; OG: obesity group, NG: normal-weight group; “t” refers to the t-statistic obtained from either a paired samples *t*-test or an independent samples *t*-test.

**Table 5:** Changes in IL-15 after 16-week combined exercise intervention.

Variable	Group	Pre	Post	*diff* (%)	Paired-t	*F*	
IL-15 (pg/mL)	OG (*n* = 15)	1.85 ± 0.91	1.39 ± 0.57	−0.45	1.700	Group	1.37
	NG (*n* = 14)	2.19 ± 0.91	1.68 ± 0.57	−0.51	1.973	Time	6.70 *
	*t-value*	1.013	1.352	−0.15		G × T	0.02

Values are *M* ± *SD*; IL-15: interleukin-15; OG: obesity group, NG: normal-weight group; “t” refers to the *t*-statistic obtained from either a paired samples *t*-test or an independent samples *t*-test; * *p* < 0.05.

## Data Availability

Data available on request due to restrictions.

## Abbreviations

The following abbreviations are used in this manuscript:

%BF	Percentage of body fat
GDF-15	Growth differentiation factor-15
IL-15	Interleukin-15
BMI	Body mass index
OMNI-RES	OMNI-Resistance Exercise Scale

## References

[1] García-Miranda A., Garcia-Hernandez A., Castañeda-Saucedo E., Navarro-Tito N., Maycotte P. (2022). Adipokines as regulators of autophagy in obesity-linked cancer. Cells.

[2] Jang S.H., Paik I.Y., Ryu J.H., Lee T.H., Kim D.E. (2019). Effects of aerobic and resistance exercises on circulating apelin-12 and apelin-36 concentrations in obese middle-aged women: A randomized controlled trial. BMC Womens Health.

[3] Alizadeh Pahlavani H. (2022). Exercise therapy for people with sarcopenic obesity: Myokines and adipokines as effective actors. Front. Endocrinol..

[4] Son J.S., Chae S.A., Park B.I., Min D., Song W. (2019). Plasma apelin levels in overweight/obese adults following a single bout of exhaustive exercise: A preliminary cross-sectional study. Endocrinol. Diabetes Nutr..

[5] Chen T.C., Huang T.H., Tseng W.C., Tseng K.W., Hsieh C.C., Chen M.Y., Chou T.Y., Huang Y.C., Chen H.L., Nosaka K. (2021). Changes in plasma C1q, apelin and adropin concentrations in older adults after descending and ascending stair walking intervention. Sci. Rep..

[6] Johann K., Kleinert M., Klaus S. (2021). The role of GDF15 as a myomitokine. Cells.

[7] Wang D., Day E.A., Townsend L.K., Djordjevic D., Jørgensen S.B., Steinberg G. (2021). GDF15: Emerging biology and therapeutic applications for obesity and cardiometabolic disease. Nat. Rev. Endocrinol..

[8] Zhang H., Fealy C.E., Kirwan J.P. (2019). Exercise training promotes a GDF15-associated reduction in fat mass in older adults with obesity. Am. J. Physiol. Endocrinol. Metab..

[9] Guo A., Li K., Xiao Q. (2020). Sarcopenic obesity: Myokines as potential diagnostic biomarkers and therapeutic targets?. Exp. Gerontol..

[10] Ahima R.S., Park H.K. (2015). Connecting myokines and metabolism. Endocrinol. Metab..

[11] Lutz C.T., Quinn L.S. (2012). Sarcopenia, obesity, and natural killer cell immune senescence in aging: Altered cytokine levels as a common mechanism. Aging.

[12] Sakuma K., Yamaguchi A. (2013). Sarcopenic obesity and endocrinal adaptation with age. Int. J. Endocrinol..

[13] O’Donoghue G., Blake C., Cunningham C., Lennon O., Perrotta C. (2021). What exercise prescription is optimal to improve body composition and cardiorespiratory fitness in adults living with obesity? A network meta-analysis. Obes. Rev..

[14] Grajek A., Sprung V.S. (2022). Global self-esteem, physical activity, and body composition changes following a 12-week dietary and physical activity intervention in older women. Int. J. Environ. Res. Public Health.

[15] Park W.I., Jung W.S., Hong K.S., Kim Y.Y., Kim S.W., Park H.Y. (2020). Effects of moderate combined resistance-and aerobic-exercise for 12 weeks on body composition, cardiometabolic risk factors, blood pressure, arterial stiffness, and physical functions, among obese older men: A pilot study. Int. J. Environ. Res. Public Health.

[16] Kim B.Y., Kang S.M., Kang J.H., Kang S.Y., Kim K.K., Kim K.B., Kim B., Kim S.J., Kim Y.H., Kim J.H. (2021). Korean Society for the Study of Obesity Guidelines for the Management of Obesity in Korea. J. Obes. Metab. Syndr..

[17] Hyun S.J., Ha S.M., Kim J.S., Kim D.Y. (2019). Effects of combined exercise program on inflammatory factors related to cardiovascular disease in elderly women. J. Korean Assoc. Phys. Educ. Sport. Girls Women..

[18] American College of Sports Medicine (2018). ACSM’s Guidelines for Exercise Testing and Prescription.

[19] Colado J.C., Furtado G.E., Teixeira A.M., Flandez J., Naclerio F. (2020). Concurrent and construct validation of a new scale for rating perceived exertion during elastic resistance training in the elderly. J. Sports Sci. Med..

[20] Matson T.E., Renz A.D., Takemoto M.L., McClure L.A., Rosenberg D.E. (2018). Physical activity participation among older adults: Trends and disparities. J. Aging Health.

[21] Scherer P.E. (2018). The multifaceted roles of adipose tissue—Therapeutic targets for diabetes and beyond: The 2015 Banting Lecture. Diabetes.

[22] Cai L., Li C., Wang Y., Mo Y., Yin J., Ma X. (2021). Increased serum GDF15 related to improvement in metabolism by lifestyle intervention among young overweight and obese adults. Diabetes Metab. Syndr. Obes..

[23] Wang D., Townsend L.K., DesOrmeaux G.J., Frangos S.M., Batchuluun B., Dumont L., Kuhre R.E., Ahmadi E., Hu S., Rebalka I.A. (2023). GDF15 promotes weight loss by enhancing energy expenditure in muscle. Nature.

[24] Eddy A.C., Trask A.J. (2021). GDF-15 and the regulation of energy balance: Emerging roles in obesity and metabolism. J. Endocrinol..

[25] Keipert S., Ost M. (2021). GDF-15 as a stress-induced myokine with metabolic regulatory function. Nat. Rev. Endocrinol..

[26] Kleinert M., Clemmensen C., Sjøberg K.A., Carl C.S., Jeppesen J.F., Wojtaszewski J.F., Kiens B., Richter E.A. (2018). Exercise increases circulating GDF15 in humans. Mol. Metab..

[27] Tchou I., Margeli A., Tsironi M., Skenderi K., Barnet M., Kanaka-Gantenbein C., Papassotiriou I., Beris P. (2009). Growth-differentiation factor-15, endoglin and NT-proBNP induction in athletes participating in an ultramarathon foot race. Biomarkers.

[28] Vinel C., Lukjanenko L., Batut A., Deleruyelle S., Pradere J.P., Le Gonidec S., Dortignac A., Geoffre N., Pereira O., Karaz S. (2018). The exerkine apelin reverses age-associated sarcopenia. Nat. Med..

[29] Sheibani S., Hanachi P., Refahiat M.A. (2012). Effect of aerobic exercise on serum concentration of apelin, TNF-α and insulin in obese women. Iran. J. Basic. Med. Sci..

[30] Ji E., Park S.J., Jang I.Y., Baek J.Y., Jo Y., Jung H.W., Lee E., Ryu D., Kim B.J. (2025). Circulating apelin levels and muscle health in older adults. J. Nutr. Health Aging.

[31] Fujie S., Sato K., Miyamoto-Mikami E., Hasegawa N., Fujita S., Sanada K., Hamaoka T., Iemitsu M. (2014). Reduction of arterial stiffness by exercise training is associated with increasing plasma apelin level in middle-aged and older adults. PLoS ONE.

[32] Kim M.S., Lee H.J., Park J.S. (2020). Effects of 12-week combined exercise on IL-15 expression and metabolic indicators in elderly women. J. Exerc. Rehabil..

[33] Hingorjo M.R., Qureshi M.A., Mehdi A. (2018). Serum interleukin-15 and its relationship with visceral fat and markers of obesity in adults. J. Pak. Med. Assoc..

[34] Tolouei Azar J., Shabkhiz F., Khalafi M. (2021). The effects of eight weeks of resistance training on serum levels of IL-15, IL-6, TNF-α, and insulin resistance in older type 2 diabetic men. J. Sport Biosci..

[35] Kim K., Kim H., Yoon M. (2018). Effects of Resistance Exercise and Fermented Soybean Consumption on Glucose Tolerance and Expressions of Immune Senescence-Related Myokines in Middle-Aged Obese Rats. J. Obes. Metab. Syndr..

